# How Well Do Commonly Used Co-contraction Indices Approximate Lower Limb Joint Stiffness Trends During Gait for Individuals Post-stroke?

**DOI:** 10.3389/fbioe.2020.588908

**Published:** 2021-01-07

**Authors:** Geng Li, Mohammad S. Shourijeh, Di Ao, Carolynn Patten, Benjamin J. Fregly

**Affiliations:** ^1^Rice Computational Neuromechanics Laboratory, Department of Mechanical Engineering, Rice University, Houston, TX, United States; ^2^Biomechanics, Rehabilitation, and Integrative Neuroscience Lab, Department of Physical Medicine and Rehabilitation, School of Medicine, University of California, Davis, Davis, CA, United States

**Keywords:** muscle co-contraction, co-contraction index, joint stiffness, electromyography (EMG), EMG-driven modeling

## Abstract

Muscle co-contraction generates joint stiffness to improve stability and accuracy during limb movement but at the expense of higher energetic cost. However, quantification of joint stiffness is difficult using either experimental or computational means. In contrast, quantification of muscle co-contraction using an EMG-based Co-Contraction Index (CCI) is easier and may offer an alternative for estimating joint stiffness. This study investigated the feasibility of using two common CCIs to approximate lower limb joint stiffness trends during gait. Calibrated EMG-driven lower extremity musculoskeletal models constructed for two individuals post-stroke were used to generate the quantities required for CCI calculations and model-based estimation of joint stiffness. CCIs were calculated for various combinations of antagonist muscle pairs based on two common CCI formulations: Rudolph et al. (2000) (*CCI*_1_) and Falconer and Winter (1985) (*CCI*_2_). *CCI*_1_ measures antagonist muscle activation relative to not only total activation of agonist plus antagonist muscles but also agonist muscle activation, while *CCI*_2_ measures antagonist muscle activation relative to only total muscle activation. We computed the correlation between these two CCIs and model-based estimates of sagittal plane joint stiffness for the hip, knee, and ankle of both legs. Although we observed moderate to strong correlations between some CCI formulations and corresponding joint stiffness, these associations were highly dependent on the methodological choices made for CCI computation. Specifically, we found that: (1) *CCI*_1_ was generally more correlated with joint stiffness than was *CCI*_2_, (2) CCI calculation using EMG signals with calibrated electromechanical delay generally yielded the best correlations with joint stiffness, and (3) choice of antagonist muscle pairs significantly influenced CCI correlation with joint stiffness. By providing guidance on how methodological choices influence CCI correlation with joint stiffness trends, this study may facilitate a simpler alternate approach for studying joint stiffness during human movement.

## Introduction

Muscle co-contraction refers to the simultaneous activation of muscles on opposite sides of a joint. It is an important mechanism used by the central nervous system to regulate joint stability (Hirokawa et al., 1991; McGill et al., 2003) and provide movement accuracy (Gribble et al., 2003; Missenard et al., 2008). Individuals who suffer from orthopedic injuries or neuromuscular disorders use elevated levels of muscle co-contraction (Lamontagne et al., 2000; Rudolph et al., 2000; Higginson et al., 2006; McGinnis et al., 2013) to generate additional joint stiffness so as to compensate for the lack of joint stability (Gollhofer et al., 1984; Kuitunen et al., 2002; Mohr et al., 2018), although evidence in support of this premise is equivocal (Banks et al., 2017). While co-contraction increases joint stiffness which in turn may improve the stability (Latash and Huang, 2015) and accuracy (Wong et al., 2009) of limb movement, it does so at the expense of increased energetic cost (Moore et al., 2014). Quantification of joint stiffness is therefore critical for understanding how this quantity adds both benefit and cost to dynamic movements such as gait.

Stroke is a common clinical condition that often impairs movement through an increase in joint stiffness (Thilmann et al., 1991; Rydahl and Brouwer, 2004; Galiana et al., 2005; Mirbagheri et al., 2008; Gao et al., 2009) and spasticity (Galiana et al., 2005; Mirbagheri et al., 2008) along with a decrease in joint range of motion (Gao et al., 2009). Some clinicians have developed rehabilitation regimens that use stretching and relaxation to help reduce joint stiffness (Bressel and McNair, 2002; Selles et al., 2005; Gao et al., 2011). Other clinicians have used assistive devices with stiffness-informed designs to help improve movement function in stroke survivors. These devices include rehabilitation robots (Vallery et al., 2008), exoskeletons (Liu et al., 2018), and ankle-foot orthoses (Singer et al., 2014). A common theme in these studies is the need for reliable quantification of joint stiffness for the design and evaluation of new treatments. However, joint stiffness is difficult to measure experimentally (Pfeifer et al., 2012) or calculate computationally, and determining it requires musculoskeletal modeling informed by appropriate muscle recruitment strategies (Sartori et al., 2015). Consequently, development of easy-to-use methods for estimating joint stiffness in a clinical setting could be valuable for improving the treatment of individuals post-stroke.

Quantification of muscle co-contraction may offer an alternative for estimating joint stiffness. Although previous studies have reported that muscle co-contraction and joint stiffness are related (Kuitunen et al., 2002; McGinnis et al., 2013; Collins et al., 2014), the relationship between these two quantities remains poorly understood, as initially noted by Hortobágyi and Devita (2000). One issue is that previous studies have quantified joint stiffness primarily in the form of quasi-stiffness. Joint quasi-stiffness is described as the gradient of the torque-angle curve rather than the true characterization of joint stiffness (Rouse et al., 2013). Since joint quasi-stiffness does not change for different levels of muscle co-contraction, it is not an accurate representation of joint stiffness generated by muscle co-contraction. From another perspective, quasi-stiffness represents the joint moment response to changes in not only joint position but also muscle activation and joint velocity (Sartori et al., 2015). To address these issues, the present study defines joint stiffness as the elastic response of a joint moment to changes in only joint position. This definition follows the recommendation of Latash and Zatsiorsky (1993) and provides a reasonable basis for the evaluation of the relationship between muscle co-contraction and joint stiffness.

The Co-Contraction Index (CCI) is a commonly used method for quantifying muscle co-contraction during human movement. Computation of a CCI involves choosing from a wide selection of methods, and previous studies have examined how differences in method affect CCI results (Knarr et al., 2012; Banks et al., 2017; Souissi et al., 2017). Two common CCI formulations (Falconer and Winter, 1985; Rudolph et al., 2000) allow clinical researchers to make a fast and easy assessment of muscle co-contraction using surface electromyographic (EMG) data, and these two formulations have been used in several studies to quantify muscle co-contraction (Kellis, 1998; Di Nardo et al., 2015; Banks et al., 2017). The selected CCI formulation with associated methodological choices could affect the extent to which the CCI is a reasonable surrogate for joint stiffness. Consequently, it would be valuable to evaluate how different methodological choices for calculating a CCI affect the CCI’s ability to approximate joint stiffness trends during activities of daily living such as gait.

This study provides a quantitative evaluation of the relationship between lower extremity muscle co-contraction indices and corresponding joint stiffnesses during gait. We calculated CCIs and lower body joint stiffnesses using EMG data collected from the lower extremity muscles of two individuals post-stroke walking at their self-selected speed. CCIs were calculated for the two common CCI formulations noted above using four different methods for EMG data post-processing. Lower body joint stiffnesses were calculated using EMG-driven musculoskeletal models calibrated using the EMG, motion capture, and ground reaction data collected from each subject. Correlations between CCIs and joint stiffnesses for each subject were calculated, and CCI calculation methods that helped improve the correlations were identified. These findings could help clinicians formulate CCIs that yield results more strongly aligned with joint stiffness trends.

## Materials and Methods

### Experimental Data

Walking data collected from two hemiparetic male subjects post-stroke were used for this study. The first subject (male, height 1.70 m, mass 80.5 kg, age 79 years, right-sided hemiparesis, lower extremity Fugl-Meyer Motor Assessment score of 32 out of a maximum 34), herein referred to as subject S1, walked at a self-selected speed of 0.5 m/s. The second subject (male, height 1.83 m, mass 88.5 kg, age 62 years, right-sided hemiparesis, lower extremity Fugl-Meyer Motor Assessment score of 25 points), herein referred to as subject S2, walked at a self-selected speed of 0.45 m/s. Both subjects walked for multiple cycles on a split-belt instrumented treadmill (Bertec Corp., Columbus, OH, United States) while motion capture (Vicon Corp., Oxford, United Kingdom), ground reaction (Bertec Corp., Columbus, OH, United States), and EMG (Motion Lab Systems, Baton Rouge, LA, United States) data were collected. EMG signals were measured at 1,000 Hz from 16 muscles in each leg (Table 1) using a combination of surface and fine-wire electrodes. For more details about the data collection and the experimental protocol, see Meyer et al. (2017). Data from ten gait cycles for each subject were selected for analysis.

**TABLE 1:** Muscles analyzed in this study.

Muscle name (abbreviation)	EMG source	Direction of moment generation			EMG scale in S1		EMG scale in S2	
		Hip	Knee	Ankle	L	R	L	R
Adductor brevis (addbrev)	Adductor longus	FLEX			0.14	0.05	0.05	0.23
Adductor longus (addlong)		FLEX			0.33	0.07	0.05	0.27
Adductor magnus distal (addmag1)		EXT			0.07	0.05	0.05	0.09
Adductor magnus ischial (addmag2)		EXT			0.05	0.05	0.05	0.31
Adductor magnus middle (addmag3)					0.07	0.05	0.05	0.25
Adductor magnus proximal (addmag4)		FLEX			0.13	0.05	0.05	0.66
Gluteus maximus superior (glmax1)	Gluteus maximus	EXT			0.32	0.20	0.43	0.09
Gluteus maximus middle (glmax2)		EXT			0.33	0.20	0.39	0.09
Gluteus maximus inferior (glmax3)		EXT			0.32	0.20	0.51	0.09
Gluteus medius anterior (glmed1)	Gluteus medius	EXT			0.71	0.62	0.06	0.92
Gluteus medius middle (glmed2)		EXT			0.69	0.63	0.06	0.92
Gluteus medius posterior (glmed3)		EXT			0.69	0.62	0.06	0.93
Gluteus minimus anterior (glmin1)					0.32	0.14	0.99	0.16
Gluteus minimus middle (glmin2)					0.30	0.15	0.99	0.16
Gluteus minimus posterior (glmin3)		EXT			0.30	0.15	0.99	0.16
Iliacus (iliacus)	Iliopsoas*	FLEX			0.05	0.05	0.05	0.05
Psoas (psoas)		FLEX			0.99	0.82	0.21	0.06
Semimembranosus (semimem)	Semimem	EXT	FLEX		0.35	0.40	0.26	0.15
Semitendinosus (semiten)		EXT	FLEX		0.30	0.40	0.26	0.15
Biceps femoris long head (bflh)	Bflh	EXT	FLEX		0.76	0.38	0.57	0.14
Biceps femoris short head (bfsh)			FLEX		0.76	0.39	0.57	0.14
Rectus femoris (recfem)	Rectus femoris	FLEX	EXT		0.48	0.27	0.65	0.05
Vastus medialis (vasmed)	Vastus medialis		EXT		0.27	0.50	0.18	0.29
Vastus intermedius (vasint)			EXT		0.31	0.44	0.16	0.28
Vastus lateralis (vaslat)	Vastus lateralis		EXT		0.32	0.11	0.16	0.27
Lateral gastronemius (gaslat)	Gasmed		FLEX	PF	0.05	0.12	0.19	0.30
Medial gastronemius (gasmed)			FLEX	PF	0.14	0.14	0.26	0.44
Tibialis anterior (tibant)	Tbialis anterior			DF	0.61	1.00	1.00	1.00
Tibialis posterior (tibpost)	Tibialis posterior*			PF	0.05	0.05	0.05	0.40
Peroneus brevis (perbrev)	Peroneus longus			PF	0.05	0.98	0.77	0.26
Peroneus longus (perlong)				PF	0.05	0.99	0.76	0.26
Peroneus tertius (pertert)				DF	0.05	0.99	0.77	0.26
Soleus (soleus)	Soleus			PF	0.65	0.96	1.00	0.08
Extensor digitorum longus^†^ (edl)	Edl*			DF	1.00	0.26		
Flexor digitorum longus^†^ (fdl)	Fdl*			PF	1.00	0.05		
Tensor fasciae latae^‡^ (tfl)	Tfl						0.52	1.00

Direction of moment generation of each muscle is indicated as FLEX – in the direction of joint flexion, EXT –in the direction of joint extension, DF – in the direction of ankle dorsiflexion, PF – in the direction of ankle plantarflexion. EMG scale is the scale factor applied to the basic EMG signal of a muscle to account for the difference between the physiological maximum and maximum observed during gait trials. S1, Post-stroke subject S1; S2, Post-stroke subject S2. *measured using fine-wire EMG. ^†^measured from only the 1st post-stroke subject (S1). ^‡^measured from only the 2nd post-stroke subject (S2).

### EMG Data Processing and EMG-Driven Model Calibration

Raw EMG data were processed using a standard methodology. The data were high-pass filtered at 40 Hz, demeaned, rectified, and low-pass filtered at a variable cutoff frequency of 3.5/period of the gait cycle (Lloyd and Besier, 2003) while using a 4th order zero phase-lag Butterworth filter (Meyer et al., 2017). Filtered EMG data were subsequently normalized to the maximum value over all cycles and resampled to 101 normalized time points for each gait cycle. Normalized EMG data for each gait cycle were offset by the minimum value so that the minimum EMG value for each gait cycle was zero. These processing procedures represent the basic approach for EMG processing adopted by other studies for the quantification of muscle co-contraction (Rosa et al., 2014). The EMG signals processed using the aforementioned procedures were defined as the basic EMG signals, *EMG*_*basic*_.

An EMG-driven modeling process was used to calibrate relevant parameters of the lower body musculoskeletal model used to represent each subject (Meyer et al., 2017). The calibrated model parameters included those defining the conversion of basic EMG into muscle excitation, muscle excitation into muscle activation via activation dynamics, and muscle activation into muscle force via a Hill-type muscle tendon model with rigid tendon. The calibration process utilized numerical optimization to adjust model parameter values so as to achieve the closest match between joint moments produced by the EMG-driven musculoskeletal model and those calculated from inverse dynamics. Conversion of basic EMG into muscle excitation involved adding an electromechanical delay and applying a muscle-specific EMG scale factor to *EMG*_*basic*_. Electromechanical delay is defined as the duration from the instant an electrical signal is received to the instant a force response is generated by the muscle. Electromechanical delay was assumed to be the same for all muscles in each leg (Meyer et al., 2017). The delays for subject S1 were 82 ms (left leg) and 93 ms (right leg, paretic side) while for subject S2 they were 100 ms (left leg) and 114 ms (right leg, paretic side). A muscle-specific scale factor (Table 1) was used to account for the difference between the estimated maximum EMG value and the maximum value over all experimental trials. The processed EMG signals resulting from calibration of both electromechanical delays and scale factors were defined as fully calibrated EMG signals, *EMG*_*calibrated*_.

To isolate the underlying effect of the two EMG parameters on quantification of co-contraction, we introduced two additional types of EMG signals: (1) scaled EMG signals *EMG*_*scaled*_, which are EMG signals normalized to the optimized maximum EMG value but without electromechanical delay, and (2) delayed EMG signals *EMG*_*delayed*_, which are electromechanically delayed EMG signals that are not normalized to the optimized maximum value. These signals were obtained as shown below:

(1) $${\text{EMG}}_{\text{scaled}}={\text{EMG}}_{\text{basic}}\times \text{scale}\,\text{factor}$$

(2) $${\text{EMG}}_{\text{delayed}}=\frac{{\text{EMG}}_{\text{calibrated}}}{\mathrm{scale}\,\mathrm{factor}}$$

### CCI Computation

CCI values were computed from processed EMG data using the two most common formulations. *CCI*_1_ was based on the formulation reported by Rudolph et al. (2000),

(3) $${\text{CCI}}_{1}\,\left(t\right)=\frac{{\text{Input}}_{L}\,\left(t\right)}{{\text{Input}}_{H}\,\left(t\right)}\,\left({\text{Input}}_{L}\,\left(t\right)+{\text{Input}}_{H}\,\left(t\right)\right)$$

while *CCI*_2_ was based on the formulation reported by Falconer and Winter (1985):

(4) $${\text{CCI}}_{2}\,\left(t\right)=\frac{2\times {\text{Input}}_{L}\,\left(t\right)}{\left({\text{Input}}_{L}\,\left(t\right)+{\text{Input}}_{H}\,\left(t\right)\right)}$$

For both formulations, *Input*_*L*_ and *Input*_*H*_ represent EMG signals from an antagonist muscle pair, where both signals were resampled to 101 normalized time points (0 – 100% of gait cycle at 1% increment). *Input*_*L*_ is the EMG signal with the lower absolute magnitude at time *t* while *Input*_*H*_ is the EMG signal with the higher absolute magnitude. For both CCI formulations, the input quantities include the four types of EMG signals (*EMG*_*basic*_, *EMG*_*scaled*_, *EMG*_*delayed*_, and *EMG*_*calibrated*_) described above. Each CCI calculation method used in this study is described by a combination of the selected CCI formulation and the selected EMG type. For example, *EMG*_*delayed*_ *CCI*_1_ means the CCI values are calculated using delayed EMG signals based on the Rudolph et al. (2000) CCI formulation.

In addition to varying the types of EMG signals used to compute CCI, this study also investigated how the difference in constituent muscles for an antagonist muscle pair could affect the relationship between CCI and joint stiffness. CCI was computed for three lower limb degrees of freedom (DOFs) in the sagittal plane: hip, knee, and ankle flexion and extension for both non-paretic side and paretic side. Lower extremity muscles were classified by their functional roles during gait (Table 1), and one muscle was selected from each of the agonist group and the antagonist group to form various combinations of antagonistic pairs. Antagonistic muscle pairs consisting of small muscles that were not major contributors to overall joint stiffness (less than 2% on average) were not included for the subsequent analyses. The majority of the EMG-based CCIs in previous studies were computed using EMG signals measured from surface muscles (Rosa et al., 2014) because the alternative fine-wire EMG method is invasive and not universally available. Therefore, despite the availability of fine-wire EMG data of deep muscles (iliacus, psoas, tibialis posterior, extensor, and flexor digitorum longus), the analyses in this study focused on CCI computed from surface EMG signals of muscles for the findings to be more applicable in clinical settings. Antagonistic muscle pairs consisting of the aforementioned deep muscles were omitted in the subsequent analyses.

### Estimation of Joint Stiffness

Sagittal plane stiffness of the lower extremity joints (hip, knee, and ankle) in each leg was estimated using a model-based formulation (Shourijeh and Fregly, 2020). The derivation starts with expressing joint stiffness as the partial derivative of joint moment *M*_*j*_ with respect to generalized coordinate *θ_*j*_* corresponding to degree of freedom (DOF) *j*:

(5) $$K_{\text{joint}}=-\frac{\partial M_{j}}{\partial \theta_{j}}$$

The net joint moment *M*_*j*_ can be expressed as the sum of the product of muscle moment arm and tendon force for each muscle spanning the joint:

(6) $$M_{j}=\sum_{i=1}^{n}r_{i\,j}\,F_{i}^{T}$$

where *r*_*ij*_ represents the moment arm of the *i*th muscle about DOF *j*, $F_{i}^{T}$ represents the tendon force of the *i*th muscle, and *n* is the total number of muscles. By substituting the expression of joint moment *M*_*j*_ into Eq. (5) and performing partial differentiation via product rule, one obtains

(7) $$K_{\text{joint}}=-\frac{\partial M_{j}}{\partial \theta_{j}}=-\sum_{i=1}^{n}\left(\frac{\partial r_{i\,j}}{\partial \theta_{j}}\,F_{i}^{T}+r_{i\,j}\,\frac{\partial F_{i}^{T}}{\partial \theta_{j}}\right)$$

Re-expressing $\frac{\partial F_{i}^{T}}{\partial \theta_{j}}$ as $\frac{\partial F_{i}^{T}}{\partial l_{i}^{M\,T}}\,\frac{\partial l_{i}^{M\,T}}{\partial \theta_{j}}$ via the chain rule and taking advantage of the fact that $r_{i\,j}=-\frac{\partial l_{i}^{M\,T}}{\partial \theta_{j}},$ where *l^*MT*^_*i*_* represents the muscle-tendon length of the *i*th muscle, *K*_*j*__*oint*_ can be expressed as the sum of the stiffness contributed by each individual muscle *K*_*mus*_ as shown below:

(8) $$K_{\text{joint}}=\sum_{i=1}^{n}K_{m\,u\,s\,i}=-\sum_{i=1}^{n}\left(\frac{\partial r_{i\,j}}{\partial \theta_{j}}\,F_{i}^{T}+r_{i\,j}^{2}\,\frac{\partial F_{i}^{T}}{\partial l_{i}^{M\,T}}\right)$$

This model-based stiffness formulation assumes that the muscle model possesses a rigid tendon. As moment arms and muscle-tendon lengths of the musculoskeletal model (Meyer et al., 2017) were represented by surrogate models in the form of polynomials of joint kinematics, muscle stiffness around a joint could be computed analytically. Identical to CCI calculations, joint stiffness was calculated at 101 normalized time points within each gait cycle.

### Statistical Analyses

The strength of association between CCI (*CCI*_1_ or *CCI*_2_) and joint stiffness *K*_*joint*_ was quantified by the Pearson correlation coefficient using the *corrcoef* function in MATLAB (MathWorks, Natick, United States). Correlation was calculated between the two time series for each of the 10 gait cycles analyzed:

(9) $$r_{1_{j}}=\text{corrcoef}\,\left(C\,C\,I_{1_{j}},K_{j}\right)$$

(10) $$r_{2_{j}}=\text{corrcoef}\,\left(C\,C\,I_{2_{j}},K_{j}\right)$$

The Wilcoxon rank sum test was performed in MATLAB using the *ranksum* function to compare the mean correlation coefficient between the two classes of data (10 pairs of correlation coefficients for 10 gait cycles). The analysis tested the null hypothesis that the two classes of data came from samples with continuous distributions possessing equal medians. The level of statistical significance was set at *p* = 0.05.

## Results

**FIGURE 1:**
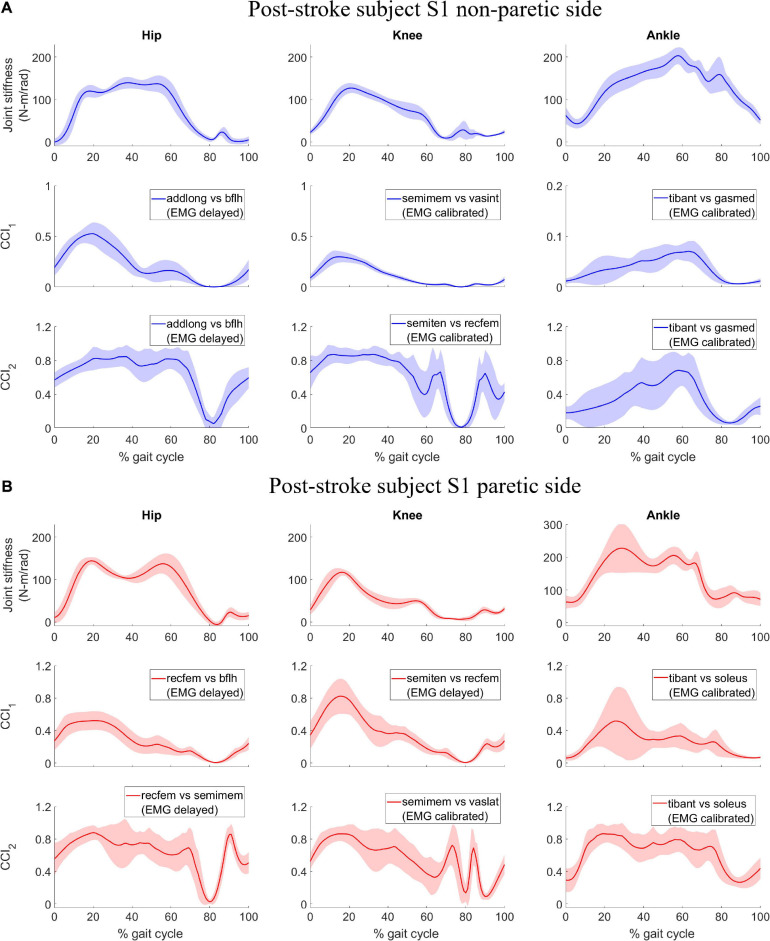
Post-stroke subject S1, lower extremity joint stiffness and sample EMG-based *CCI*_1_ and *CCI*_2_ values (mean ± 1 standard deviation) for **(A)** non-paretic side, and **(B)** paretic side. The sample EMG-based CCIs were selected for display because of their highest correlation with corresponding joint stiffness. The antagonistic pair of muscles selected for CCI computation are identified and EMG signal type is displayed in parenthesis.

Joint stiffness trends were mostly symmetrical between the non-paretic and paretic side for subject S1 (Figures 1A,B, 1st row). For the hip joint on each side, joint stiffness increased steadily in the early stance phase (0 – 15% gait cycle), then were largely maintained at a constant level slightly above 100 N-m/rad for the remainder of the stance phase (15 – 55% gait cycle), then decreased during late stance and swing phases (55 – 100% gait cycle). For the knee joint on each side, joint stiffness increased steadily early in the stance phase (0 – 20% gait cycle) and then gradually decreased from the peak value. For the ankle joint, however, joint stiffness on the paretic side peaked at a magnitude much higher than that on the non-paretic side at approximately 30% gait cycle. The decline in joint stiffness was more gradual on the non-paretic side during swing phase than what was more sudden on the paretic side.

**FIGURE 2:**
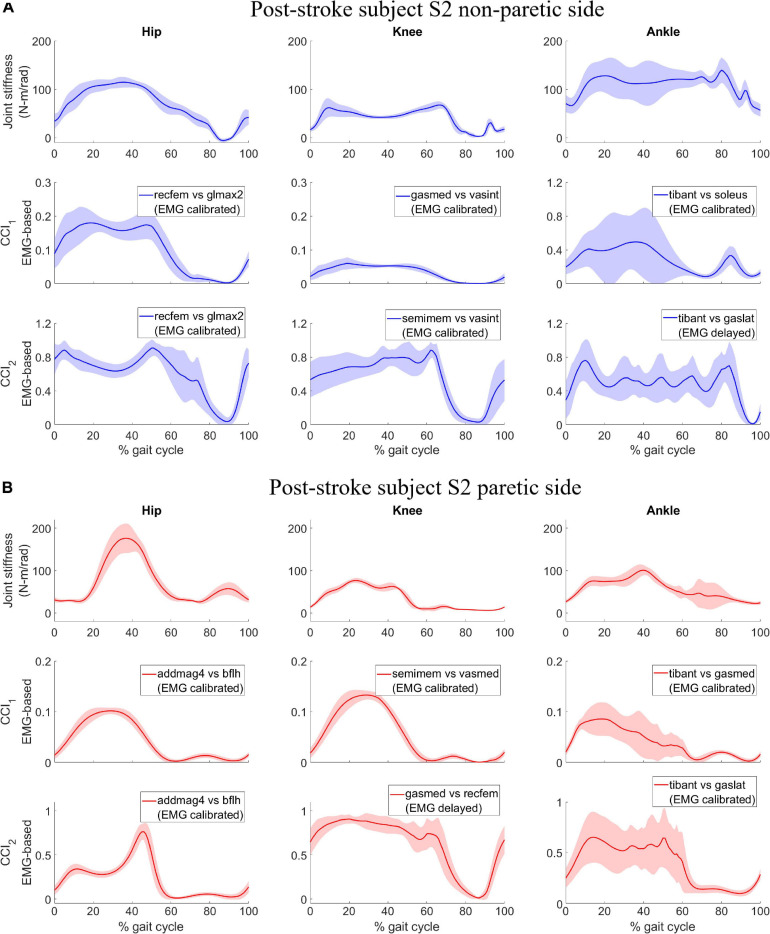
Post-stroke subject S2, lower extremity joint stiffness and sample EMG-based *CCI*_1_ and *CCI*_2_ values (mean ± 1 standard deviation) for both **(A)** non-paretic side, and **(B)** paretic side. The sample EMG-based CCIs were selected for display because of the highest correlation with corresponding joint stiffness. The antagonistic pair of muscles selected for CCI computation are identified and EMG processing method is displayed in parenthesis.

In contrast, joint stiffness trends were asymmetrical between the non-paretic and paretic side for subject S2 (Figures 2A,B, 1st row). Joint stiffness for hip, knee, and ankle on the non-paretic side was sustained at a high level to a much later point in the gait cycle before declining than that on the paretic side. The joint stiffness trends coincided with the subject’s gait pattern which had both longer than normal stance phase on the non-paretic side (0 – ∼75% gait cycle) and shorter than normal stance phase on the paretic side (0 – ∼50% gait cycle). Also observed from joint stiffness trends of subject S2 was that joint stiffness for the hip on the paretic side reached a peak magnitude much higher than that on the non-paretic side at 35% gait cycle and was followed by a sharp decline which was not seen on the paretic side.

**FIGURE 3:**
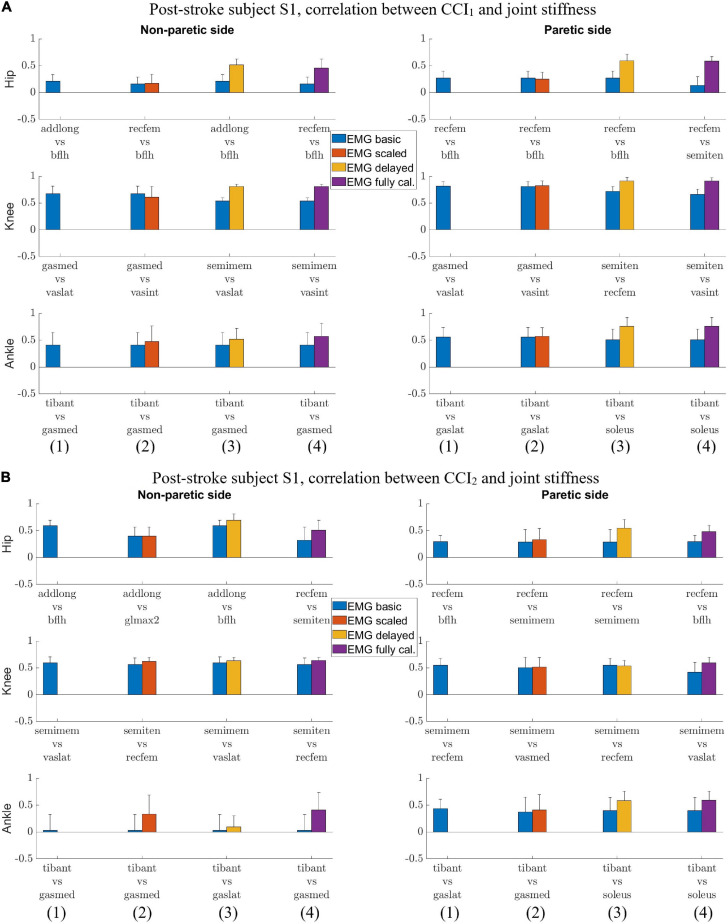
**(A)** Post-stroke subject S1, correlation between *CCI*_1_ and joint stiffness *K*_*joint*_. Bars are at the mean value of the Pearson correlation coefficient, and error bars are at one standard deviation (+/– depending on the sign of mean value). Each muscle combination for antagonistic pairing displayed in the figure represents the best correlation between *K*_*joint*_ and *CCI*_1_ computed using a specific type of EMG signals: (1) *EMG*_*basic*_ (blue); (2) *EMG*_*scaled*_ (red); (3) *EMG*_*delayed*_ (yellow); and (4) *EMG*_*calibrated*_ (purple). **(B)** Post-stroke subject S1, correlation between *CCI*_2_ and joint stiffness *K*_*joint*_. Bars are at the mean value of the Pearson correlation coefficient and error bars are at one standard deviation (+/– depending on the sign of mean value). Each muscle combination for antagonistic pairing displayed in the figure represents the best-in-class correlation between *K*_*joint*_ and *CCI*_2_ computed using a specific type of EMG signals: (1) *EMG*_*basic*_ (blue); (2) *EMG*_*scaled*_ (red); (3) *EMG*_*delayed*_ (yellow); and (4) *EMG*_*calibrated*_ (purple).

For subject S1, we observed correlation ranged from moderate to strong between *CCI*_1_ and joint stiffness (Figure 3A) and from weak to moderate between *CCI*_2_ and joint stiffness (Figure 3B). Correlation between *CCI*_1_ and *K*_*joint*_, r_1_ was moderate (0.5 < ${\bar{r}}_{1}$ < 0.7) for the hip joint, strong (${\bar{r}}_{1}$ > 0.7) for the knee joint, and moderate (0.5 < ${\bar{r}}_{1}$ < 0.7) for the ankle joint on both sides. Correlation strength was assessed based on Moore et al. (2015). Correlation between *CCI*_2_ and *K*_*joint*_, r_2_ were moderate (0.5 < ${\bar{r}}_{2}$ < 0.7) for the hip joint on both sides, moderate (0.5 < ${\bar{r}}_{2}$ < 0.7) for the knee joint on both sides, weak (0.3 < ${\bar{r}}_{2}$ < 0.5) for the ankle joint on the non-paretic side and moderate (0.5 < ${\bar{r}}_{2}$ < 0.7) on the paretic side.

**FIGURE 4:**
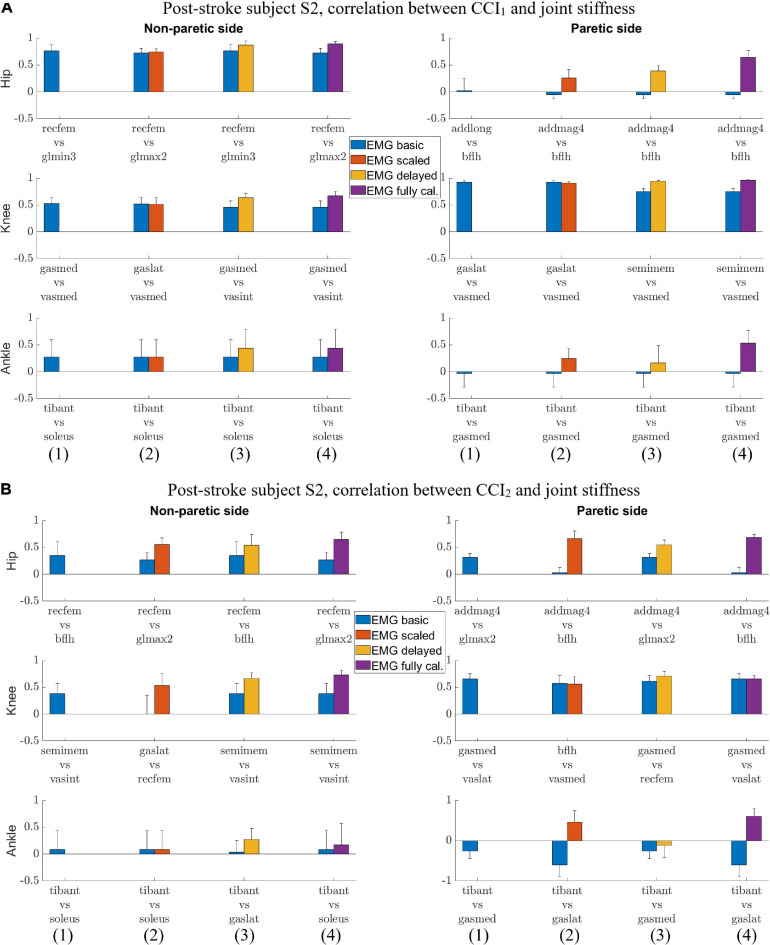
**(A)** Post-stroke subject S2, correlation between *CCI*_1_ and joint stiffness *K*_*joint*_. Bars are at the mean value of the Pearson correlation coefficient, and error bars are at one standard deviation (+/– depending on the sign of mean value). Each muscle combination for antagonistic pairing displayed in the figure represents the best correlation between *K*_*joint*_ and *CCI*_1_ computed using a specific type of EMG signal: (1) *EMG*_*basic*_ (blue); (2) *EMG*_*scaled*_ (red); (3) *EMG*_*delayed*_ (yellow); and (4) *EMG*_*calibrated*_ (purple). **(B)** Post-stroke subject S2, correlation between *CCI*_2_ and joint stiffness *K*_*joint*_. Bars are at the mean value of the Pearson correlation coefficient, and error bars are at 1 standard deviation (+/– depending on the sign of mean value). Each muscle combination for antagonistic pairing displayed in the figure represents the best correlation between *K*_*joint*_ and *CCI*_2_ computed using a specific type of EMG signal: (1) *EMG*_*basic*_ (blue); (2) *EMG*_*scaled*_ (red); (3) *EMG*_*delayed*_ (yellow); and (4) *EMG*_*calibrated*_ (purple).

For subject S2, we observed correlation ranged from weak to strong between *CCI*_1_ and joint stiffness (Figure 4A) and from weak to strong between *CCI*_2_ and joint stiffness (Figure 4B). Correlation between *CCI*_1_ and *K*_*joint*_, r_1_ was strong (${\bar{r}}_{1}$ > 0.7) for the hip joint on the non-paretic side and moderate (0.5 < ${\bar{r}}_{1}$ < 0.7) on the paretic side, moderate (0.5 < ${\bar{r}}_{1}$ < 0.7) for the knee joint on the non-paretic side and strong (${\bar{r}}_{1}$ > 0.7) on the paretic side, weak (0.3 < ${\bar{r}}_{1}$ < 0.5) for the ankle joint on the non-paretic side and moderate (0.5 < ${\bar{r}}_{1}$ < 0.7) on the paretic side. Correlation r_2_ between *CCI*_2_ and *K*_*joint*_ were moderate (0.5 < ${\bar{r}}_{2}$ < 0.7) for the hip joint on both sides, strong (${\bar{r}}_{2}$ > 0.7) for the knee joint on both sides, weak (0.3 < ${\bar{r}}_{2}$ < 0.5) for the ankle joint on the non-paretic side and moderate (0.5 < ${\bar{r}}_{2}$ < 0.7) on the paretic side.

**FIGURE 5:**
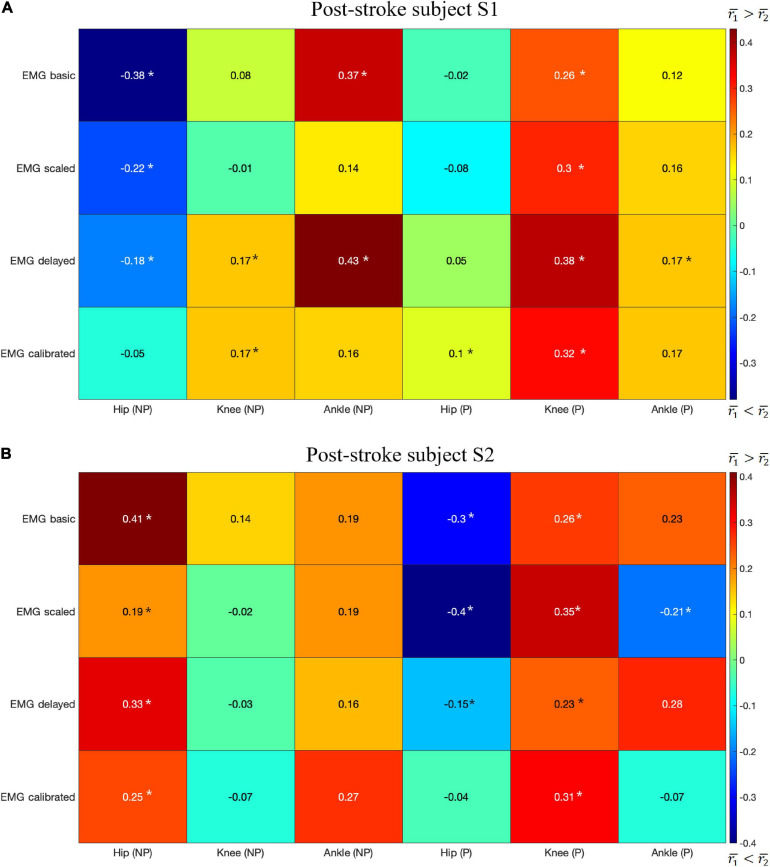
The difference in the highest mean correlation between *CCI*_1_ and *K*_*joint*_ ($\bar{r_{1}}$) and between *CCI*_2_ and *K*_*joint*_ ($\bar{r_{2}}$) for each type of EMG signal. Positive difference (red) indicates *CCI*_1_ has a higher correlation with *K*_*joint*_ than does *CCI*_2_, while negative difference (blue) indicates the opposite. NP, non-paretic side, P, paretic side. The results are for: **(A)** Post-stroke subject S1, and **(B)** Post-stroke subject S2. A star (*) indicates a statistically significant Wilcoxon rank sum test result (*p* < 0.05).

The highest mean values for *r*_1_ were generally higher than those for *r*_2_ for both subjects with a few exceptions (Figures 5A,B). Correlations *r*_1_ and *r*_2_ were evaluated at six joints for both subjects, which yielded a total of 12 cases for comparing *r*_1_ and *r*_2_. In 7 of the 12 cases, *r*_1_ was larger than *r*_2_ (Figures 5A,B): S1 Knee (NP) Ankle (NP) Knee (P) Ankle (P), S2 Hip (NP), Ankle (NP), and Knee (P), where NP refers to the non-paretic side and P refers to the paretic side. In only 3 of the 12 cases was *r*_2_ clearly higher than *r*_1_ (Figures 5A,B): S1 Hip (NP), S2 Hip (P), Ankle (P). In the other two case, S1 Hip (P) and S2 Knee (NP), neither *r*_1_ nor *r*_2_ was clearly higher than the other.

We also identified the EMG processing methods and antagonistic muscle pairings that would likely yield the highest correlations between the CCIs and joint stiffness. The CCI with highest correlation to joint stiffness at each joint for both subjects was calculated based on either *EMG*_*delayed*_ or *EMG*_*calibrated*_ (Figures 3A,B, 4A,B). CCIs calculated using *EMG*_*scaled*_ did not always yield higher correlations with joint stiffness than did those calculated using *EMG*_*basic*_. The antagonist muscle pairs that yielded that highest correlation between CCIs and joint stiffness (Figures 3A,B, 4A,B) were: 1. Adductors-hamstrings or quadriceps-hamstrings combinations for the hip joints; 2. Quadriceps-hamstrings combinations for the knee joints; 3. Tibialis anterior-gasctronemii or tibialis anterior-soleus combinations for the ankle joints.

## Discussion

This study evaluated how well different CCI formulations approximate lower extremity joint stiffness trends during gait for individuals post-stroke. Joint stiffness trends may help reveal gait pathologies in these individuals as demonstrated in this study. In addition, joint stiffness may potentially be used to improve the design of rehabilitation treatments and assistive devices for individuals post-stroke. However, the difficulty of measuring or computing joint stiffness is well documented. It would therefore be beneficial to the clinical community if commonly used co-contraction indices correlated well with joint stiffness, thereby providing easy-to-calculate surrogate measures of joint stiffness. Although moderate to strong correlation was observed between some CCI formulations and corresponding joint stiffness, this correlation was highly dependent on the methodological choices made for CCI computation. The conditions under which we observed the highest CCI correlations with joint stiffness were obtained can be summarized as follows: (1) *CCI*_1_ formulation (Rudolph et al., 2000) was better than *CCI*_2_ formulation (Falconer and Winter, 1985); (2) EMG signals with calibrated electromechanical delay (*EMG*_*delayed*_ and *EMG*_*calibrated*_) worked better than did *EMG*_*basic*_ or *EMG*_*scaled*_ when calculating *CCI*_1_, (3) Some antagonist muscle pairs worked better than did other antagonist muscle pairs when calculating *CCI*_1_. These findings could be used as a preliminary foundation for predicting joint stiffness trends from EMG-based measurement of muscle co-contraction.

Joint stiffness trends can help reveal gait pathologies as demonstrated in this study. On the surface, the joint stiffness trends confirmed clinical observations about the post-stroke subjects studied. Subject S1 has relatively high motor functioning post-stroke (Fugl-Meyer Motor Assessment score: 32 points), and joint stiffness trends between both non-paretic and paretic sides were symmetrical to a certain extent just as the gait patterns were. Subject S2 has relatively low motor functioning (Fugl-Meyer Motor Assessment score: 25 points) and gait asymmetry is a direct consequence. This was observed as longer than normal stance phase on the non-paretic side and shorter than normal stance phase on the paretic side, indicating possibly a compensation from the non-paretic side for weakness on the paretic side. The gait asymmetry observed was well supported by the trends of joint stiffness we estimated. Delving deeper into the gait pathologies, both subjects experienced a sudden spike in joint stiffness at joints on the paretic side: S1 (ankle) and S2 (hip). The former incidence was due to the abnormally high activation of the soleus muscle, which was not observed on the non-paretic side. The latter incidence was due to the abnormal activation of hip flexors in iliacus and psoas, compounded by the unexpectedly large stiffness generation by adductors, which are not conventionally considered as major hip flexors. The model-based estimation of joint stiffness, and calculated CCIs to some extent, may offer a way to probe into the root cause of these pathologies, providing valuable knowledge to both diagnostics and treatment of gait pathologies for stroke survivors.

Co-Contraction Index would be a suitable candidate to consider for addressing the aforementioned clinical needs because of its common adoption in the clinical community and simplicity in usage. Various methodological choices in CCI calculation were explored in this study. One key decision was to not include moment-based CCI and instead focus on EMG-based CCI. Even though moment-based CCI explored in previous studies (Knarr et al., 2012; Souissi et al., 2017) may achieve a stronger correlation with joint stiffness, as a product from advanced neuromusculoskeletal modeling and simulation, the moment-based CCI is considered to be unfit to the aim of this study, which is to build the preliminary knowledge of using some tools that can be readily deployed in the clinical setting to approximate joint stiffness. EMG-based CCI would on the other hand represent a more viable option because of its simplicity and common adoption. As this study focused on EMG-based CCI, key methodological choices in CCI calculation that would help improve correlation with joint stiffness would be identified in the following discussion.

We compared the correlation between CCIs and joint stiffness for each of the six lower extremity joints in both legs of two subjects. The comparison shows that correlation between *CCI*_1_ and joint stiffness *K*_*joint*_ (*r*_1_) was generally higher than for *CCI*_2_ (*r*_2_) in more cases if each comparison at a joint on one of the subjects was considered one case (Figures 5A,B). *r*_1_ was higher than *r*_2_ in 7 out of the 12 total cases, but lower in 3 other cases: S1 Hip (NP), S2 Hip (P), and Ankle (P). *K*_*joint*_ is a sum of the stiffness generated by all the individual muscles *K*_*mus*_. The sum term in the *CCI*_1_ formulation, *Inupt*_*L*_+*Input*_*H*_, was more effective at characterizing this summation than any term in the *CCI*_2_ formulation. This effectiveness became more pronounced when the quantities used for CCI computation from each muscle were accurate proxies for the corresponding *K*_*mus*_. On the other hand, the *CCI*_2_ formulation was more suitable for quantifying the ratio of antagonist muscle activities. This is demonstrated by the observation that the correlation between *CCI*_2_ and *K*_*joint*_ was comparable to the correlation between the *Input*_*L*_ / *Input*_*H*_ term of *CCI*_1_ and *K*_*joint*_. Close examination also showed that *CCI*_2_ values for subject S1 reached a peak in magnitude in the swing phase (∼60 to 100% gait cycle) comparable to that during the stance phase (0 to ∼60% gait cycle) at several joints (Figures 1A,B). This phenomenon was deemed unlikely to be physiological. This exposes the limitation of the *CCI*_2_ formulation that when the two quantities from antagonist muscles become close in magnitude, *CCI*_2_ would report a high level of co-contraction regardless of how small both quantities might be, as *CCI*_2_ focuses on quantifying the ratio between the two quantities. Since the *CCI*_1_ formulation was a better choice than *CCI*_2_ for approximating joint stiffness trends, the subsequent discussion will focus on the methodological choices involved in calculating *CCI*_1_.

Electromyography processing methods would affect the correlation between EMG-based CCIs and joint stiffness. From the *EMG*_*basic*_ signals, two modifications were applied to obtain the other types of processed EMG signals. One modification was adding electromechanical delay from *EMG*_*basic*_ to *EMG*_*delayed*_. This modification increased the correlation between *CCI*_1_ and *K*_*joint*_. EMG signals are able to convey partial information about joint stiffness because of the relationship between EMG amplitude and *F*_*mus*_. Introducing electro-mechanical delay improves the synchronization between an EMG signal the resulting muscle force. Consequently, the correlation between the EMG signal and *K*_*mus*_ increases, resulting in an increased correlation between the *EMG*_*delayed*_-based *CCI*_1_ and *K*_*joint*_. The second modification was applying a muscle-specific scale factor (Table 1), i.e., from *EMG*_*basic*_ to *EMG*_*scaled*_ and from *EMG*_*delayed*_ to *EMG*_*calibrated*_. This modification did not produce a clear improvement in the correlation between resultant *CCI*_1_ and *K*_*joint*_. Applying the scale factor did not change the ability of the EMG signals to represent *K*_*mus*_, and the correlation between scaled EMG signal and *K*_*mus*_ remained the same as before scaling. However, the muscle-specific scale factor did change the relative contribution of muscle EMG amplitudes to the sum term in the *CCI*_1_ formulation, *Input*_*L*_+ *Input*_*H*_. In some cases, a change in the sum term caused a decrease in the correlation between resultant *CCI*_1_ and *K*_*joint*_. Although applying muscle-specific scale factors changed muscle force estimates during the calibration of the EMG-driven musculoskeletal model, these scale factors did not consistently increase the correlation between *CCI*_1_ and *K*_*joint*_. Because of a definite improvement in correlation with *K*_*joint*_ from having the electromechanical delay, *EMG*_*delayed*_-based and *EMG*_*calibrated*_-based CCIs both yield higher correlation with *K*_*joint*_ than the other EMG-based CCIs. Despite yielding comparable level of correlation with *K*_*joint*_, *EMG*_*delayed*_-based *CCI*_1_ is more aligned with our goal than *EMG*_*calibrated*_-based for approximating joint stiffness trends using tools readily available in clinical settings, because while the electromechanical delay for both EMGs can be measured experimentally, *EMG*_*delayed*_ does not require model-based calibration of the scale factors but *EMG*_*calibrated*_ does require that.

This study explored various combinations of antagonistic muscle pairing for computing CCIs. We identified that CCIs computed from the following combinations would likely have higher correlation with the joint stiffness than the others: adductors-hamstrings or quadriceps-hamstrings for the hip joints; quadriceps-hamstrings for the knee joints; tibialis anterior-gasctronemii or tibialis anterior-soleus for the ankle joints. We compared these combinations with the ones in the literature to see if we have identified ones that are less commonly used. For the hip joint, we could only find one study in which CCI was computed (Hoang et al., 2019), which was moment-based and used a formulation different than the two equations examined in this study. Our study found that EMG from conventional hip flexor-extensor combination, e.g., rectus femoris-biceps femoris long head or rectus femoris-semitendinosus could yield a moderate correlation with hip flexion-extension joint stiffness. We also found the more unconventional adductor-biceps femoris long head combination could present another option to yield a moderate correlation between EMG-based CCI and joint stiffness. For the knee joint, there are two commonly used combinations in the literature: quadriceps-hamstrings (Kellis et al., 2003; McGinnis et al., 2013; Mohr et al., 2018), and quadriceps-gastrocnemii (Rudolph et al., 2000; Lewek et al., 2006; Mohr et al., 2018). Our study found that antagonistic pairs formed from the quadriceps-hamstrings combination is better than the quadriceps-gastrocnemii combination for achieving high correlation between CCI and knee joint stiffness. For the ankle joint, our study found that tibialis anterior-gastrocnemii or tibialis anterior-soleus combinations could yield a moderate correlation between CCI and ankle joint stiffness. The combinations were consistent with the commonly used in the literature (Böhm and Hösl, 2010; Di Nardo et al., 2015). Our study identified the antagonistic muscle pairings used in literature that would yield a correlation between CCI and joint stiffness, and also discovered some alternative options that were less conventional.

Contrary to the general trend noted above that the correlation between *CCI*_1_ and joint stiffness *K*_*joint*_ was generally higher than for *CCI*_2_, some discrepancies existed: (1) at the hip joint on the non-paretic side of subject S1; (2) at the hip joint on the paretic side of subject S2; and (3) at the ankle joint on the paretic side of subject S2, where *CCI*_2_ was better correlated with *K*_*joint*_. These discrepancies are possibly because this study presented CCI data of only the muscles from which EMG data could be obtained by surface measurement. Although fine-wire EMG data for some muscles were also collected from the subjects studied to be used for EMG-driven model calibration and estimation of joint stiffness, these muscles were excluded from the CCI analysis, since EMG data would not be available from them in a clinical setting. Among the muscles omitted were iliacus and psoas, two primary hip flexors; and extensor digitorum longus and tibialis posterior, one primary dorsiflexor, and one primary plantarflexor, respectively. These discrepancies could be rectified if the EMG data from the omitted muscles were made available for CCI calculation. Even though invasive, the fine-wire EMG measurement technique could still provide valuable information for the estimation of muscle co-contraction and joint stiffness when such measurements were allowed.

This study also found that the correlation between CCIs and joint stiffness is generally lower at the ankle joints than at other lower body joints for both subjects. It is possibly because a complex joint in the likes of ankle is actuated by a relatively small number of muscles in the musculoskeletal models (Subject S1: 3 dorsiflexors and 7 plantarflexors; Subject S2: 2 and 6, respectively). These muscles actuate motion in the subtalar inversion-eversion DOF in addition to the dorsi or plantar flexion. It is difficult to allocate the precise amount of muscle activation to the actuation of ankle joint in the sagittal plane. Therefore, the comparison between muscle co-contraction and joint stiffness is skewed. Moreover, we chose to omit muscle activities from extensor digitorum longus and tibilais posterior because they were obtained through fine-wire EMG and thus were not suitable for the goals of this study. This decision limited the number of muscles available for representing co-contraction around ankle joint, limiting our options to identifying muscle activities in synchronization with the generation of joint stiffness, hence resulting in a relatively lower correlation between CCIs and joint stiffness at the ankle joints than at the other joints.

One limitation of this study was that the joint stiffness used for comparison with different CCI methods was obtained from a neuromusculoskeletal model instead of experimentally using a joint perturbation technique. A model-based approach was used since the perturbation approach is difficult to implement experimentally, especially for dynamic tasks such as gait (Pfeifer et al., 2012). Consequently, there are very few reports of experimental measurements, especially during dynamic tasks, and they are only preliminary (Kobayashi et al., 2010; Shorter et al., 2019). The model-based approach has been reported to generate joint stiffness estimates that compare well with experimental joint stiffness measurements for isometric conditions (Pfeifer et al., 2012; Sartori et al., 2015). A published model for estimating joint stiffness (Shourijeh and Fregly, 2020) was used in the present study, and the model parameters were calibrated using a validated EMG-driven modeling process (Meyer et al., 2017). Thus, the model closely reproduced the subject’s experimental joint moments when the subjects’ experimental EMG and kinematic data were used as input, suggesting that the estimated muscle forces and thus joint stiffness values are likely to be at least reasonable. Our model-predictions of joint stiffness are generally consistent with the limited published results (Pfeifer et al., 2012; Sartori et al., 2015). The trends in the model estimates of joint stiffness were also supported by clinical observation as previously discussed. Ideally, if a system that can easily measure joint stiffness in vivo during different activities is developed in the future, it can provide great benefits to the clinical and research community, including direct data to evaluate the models used to estimate joint stiffness.

Another limitation of this study was that it analyzed gait data collected from only two hemiparetic subjects. Although the collection of sixteen channels (including six fine-wire) of EMG from each leg of the subject during walking was time-consuming and not a common practice, it facilitated the calibration of our musculoskeletal model. Although analyzing two subjects limits our ability to draw more general conclusions that could be applied to the stroke population, at the same time, this dataset provided a unique opportunity to build a musculoskeletal model of the subjects and calibrate the model parameters using an EMG-driven framework that did not require prediction of any missing EMG signals as in Sartori et al. (2014). Despite the relatively small number of subjects, the subjects of this study covered a wide spectrum in the post-stroke population, as one maintained relatively high motor functioning abilities while the other was more impaired in motor functioning abilities. The two subjects also exhibited different pathologies during gait. One had abnormal activation in soleus muscle while the other had abnormal activation in hip flexors. These different pathologies provide unique opportunities and testing cases to evaluate the premise of this study. Future work of the current study would repeat the analysis with data from more subjects post-stroke to be able to make more generalizable conclusions and possible recommendations to the clinicians.

In conclusion, this study demonstrated the feasibility of using EMG-based CCIs to approximate lower limb joint stiffness trends during gait for individuals post-stroke. A number of methodological choices for CCI computation were examined. Key methodological choices to achieve the highest possible correlation between CCI and joint stiffness should include the use of *CCI*_1_ formulation and adding calibrated electromechanical delay to the EMG signals for computing EMG-based *CCI*_1_. Antagonistic muscle pairings that yielded the highest correlations between CCI and joint stiffness were also identified. These findings provide the preliminary knowledge to help clinicians formulate CCI that may yield results more aligned with joint stiffness trends during gait for individuals post-stroke. By using CCI to approximate joint stiffness trends, this study may open an alternative approach to estimate joint stiffness, which is difficult to obtain through either computational modeling or experimental measurement.

## Data Availability Statement

The experimental data, OpenSim models, and Matlab code used to perform this study are available at https://simtk.org/projects/ccivsjointstiff.

## Ethics Statement

All experimental procedures were approved by the University of Florida Health Science Center Institutional Review Board (IRB-01) and the subjects provided written informed consent prior to participation.

## Author Contributions

GL, DA, MS, and BF: conceptualization, investigation, methodology, and formal analysis. BF: funding acquisition. BF and CP: data collection. MS and BF: supervision. GL, MS, BF, and CP: drafting the manuscript. GL, DA, MS, BF, and CP: revising the manuscript. All authors contributed to the article and approved the submitted version.

## Conflict of Interest

The authors declare that the research was conducted in the absence of any commercial or financial relationships that could be construed as a potential conflict of interest.
